# Teaching Parents via Online Asynchronous Training to Use Speech-Generating Devices with Their Autistic Children: A Pilot Study

**DOI:** 10.3390/children11101194

**Published:** 2024-09-29

**Authors:** Lauren Fischbacher, Robin L. Dodds, Ingrid Shiyin Tien

**Affiliations:** 1 Division of Human Development and Psychology, University of California, Los Angeles, Moore Hall, 457 Portola Plaza, Los Angeles, CA 90095, USA; lfischb@g.ucla.edu (L.F.); istien@g.ucla.edu (I.S.T.); 2 Division of Special Education & Counseling, California State University, Los Angeles, King Hall, 5151 State University Dr, Los Angeles, CA 90032, USA

**Keywords:** parent training, early intervention, naturalistic, parent-mediated interventions, parent responsiveness, telepractice, communication support needs, autism, nonspeaking

## Abstract

**Background/Objectives:** Telepractice interventions have been found to alleviate barriers families face when seeking communication interventions. This study is a multiple-baseline single-subject design that measures parent communication opportunities and parent responsiveness to determine if parent training through online modules created for parents of children with communication support needs can be effective for training parents of autistic children with communication support needs. **Methods:** This study replicates work by utilizing online training used as well as the same variables and definitions. This study expands the original study by providing the children with speech-generating devices (SGDs). SGDs are an assistive technology tool to increase language production and give access to language to minimally verbal autistic people. A central difference between this study and study is that the only training parents received was the online modules and written instructions to set up the SGD. **Results:** Overall, the POWR modules appear to positively impact the communication opportunities provided by the parent during play and activities, increase child communication, and improve parent proficiency in implementing the POWR strategy. **Conclusions:** There is a need for a larger single-case study or a randomized control trial to replicate these findings. Additional instruction may be needed for parents of children with autism around responsive interactions. This study adds to innovative ways of providing family-centered training and access to AAC for those with barriers to service.

## 1. Online Modules to Teach Parents with Autistic Children to Implement an AAC Communication Strategy

Approximately 30% of autistic children are nonspeaking or speak minimally [1]. Early interventions and support are essential for this population to see gains in communication and social skills [2]. The POWR parent communication training for alternative and augmentative communication (AAC) using online modules is a promising solution with a growing evidence base for children with communication support needs, which can help reduce barriers to services for parents and families [3]. Additionally, The POWR strategy aligns with the social and communication needs of autistic children [4,5]. The POWR modules are a series of pre-recorded videos and easily digestible sections of written text to teach the POWR method to caregivers to use AAC in the home. The POWR method consists of four easy-to-remember steps—(1) prepare the activity and AAC; (2) offer an opportunity for communication; (3) wait for the child’s communication; and (4) respond to the child’s communication [3]. These steps are taught over six short modules that contain videos, activities, and quizzes. The strategy is designed to be used during planned activities, like play using specific toys, art activities, or music-making activities [3].

This preliminary study aims to determine the effectiveness of the modules on their own when paired with a speech-generating device (SGD) for parents with children with and suspected to have autism and who also have communication support needs. In previous studies that used the POWR strategy, trainees took the modules and received additional one-on-one training in the clinic until the adult reached fidelity using the POWR strategy [3,4]. Additionally, not all participants in the studies used an SGD as their AAC. AAC is all communication that is not speech—e.g., sign, gesture, pointing, communication board, or an SGD. The previous study included participants with a variety of different AAC modalities. The present study omits the in-clinic training in order to explore if the online modules alone are enough to see the effectiveness of the modules without additional training. This study also provided an SGD to all participants and examined the POWR module as a framework for introducing an SGD.

### 1.1. Parent and Caregiver Communication Training for Autism

The introduction of AAC and its initial use can be challenging for families. Cultural factors, such as language or communication barriers, may contribute to negative feelings [6], and caregivers will likely need training and support for years as their child progresses through school [6,7]. However, given the proliferation of mobile technology (i.e., smartphones and tablets) over the last decade, parents of young children are now more receptive to symbol-based communication apps (i.e., SGDs) and feel less stigma and negative feelings about AAC than in previous years [7].

Training is imperative for families to incorporate the AAC device in their homes. Children whose parents participate in AAC training or use AAC with their children during everyday activities show more significant improvements in spontaneous communication and social skills than children whose caregivers do not receive training [8,9]

Obtaining access to an AAC system and training in its use is challenging for many families due to the difficulties they may face in procuring assessment and funding for a device, locating an experienced AAC provider, and scheduling training in their busy lives [6,7]. However, it is clear that early access to AAC leads to greater improvements in communication and social interaction for children with ASD [10]. For families that live in underserved areas with limited time and resources or families in rural areas who have difficulty traveling, this often means that children are missing out on crucial early support and services for their language development. Providing online training and remote access can bring more equitable access to AAC training for children and families by minimizing these barriers [11]. Furthermore, the flexibility associated with remote models is endorsed by both practitioners and parent [12]. Live and pre-recorded online modules allow access to training materials for an extended period so that they can master the use of the AAC device and techniques for implementation in daily activities [3].

### 1.2. Utilizing POWR + SGD with Autistic Children

The key elements of the POWR intervention that make it an ideal intervention for autistic children with communication support needs are utilizing an AAC system, such as an SGD (speech-generating device), naturalistic elements, and parent responsiveness. Naturalistic interventions, SGDs, and parent-implemented interventions are all considered evidence-based practices for autism [13].

AAC appears to have an impact on many behavior categories for autistic children including communication, social skills, challenging behaviors, and spelling—however, the strongest evidence base shows improvements in communication [14]. Parent–child relationships and conversations are important for language development and developmentally targeted parent–child conversations play a vital role in the development of language skills [15,16]. Parents are widely considered children’s first language teachers. However, parents of children with communication support needs are less likely to spend time with their children in parent–child interactions [8,17], In addition, parents may be less responsive to their child’s communication, are more direct when communicating, and use fewer language learning support strategies [18]. Salmon and colleagues [16] propose that the parent–child conversation has a direct relationship with language ability, psychopathology, self-regulation, and emotional understanding in children. Children who do not participate in parent–child conversations, such as children with ASD and communication support needs, are potentially vulnerable.

Parent responsiveness, a critical element in parent-mediated interventions for young children and infants with communication delays [18], has shown promise by improving child social interaction and communication behaviors [19,20], Parent-responsiveness and responsive teaching is linked to positive outcomes in many developmental domains: communication (e.g., joint activity, vocalization), cognition (e.g., social play, exploration), social–emotional functioning (e.g., trust, self-regulation), and motivation (e.g., persistence, enjoyment) [21], Parent responsiveness strategies are best taught in naturalistic settings, such as in the child’s home doing normal daily activities, to aid in generalization [22].

Parent verbal responsiveness (PVR), particularly, responding to a child’s communication and a child’s behavior, are both predictors of language for autistic young children [23,24] and have been found to be value-added predictors of expressive and receptive spoken language growth for autistic children [25], Substantial empirical evidence associates PVR with autistic children’s communication in two areas—focus of attention and communication acts [23]. Due to AAC’s amplified demand for attention compared with other forms of communication [26], parent responsiveness, in particular PVR, is arguably an ideal strategy for an AAC intervention.

### 1.3. The Present Study

This study sought to understand the efficacy of remote learning in training parents to implement a parent-mediated AAC communication intervention for parents with autistic children. The AAC system that was utilized for this study was an SGD– an Amazon Kindle table with Coughdrop, an open-source AAC application [27]. The goal was to provide the basis of a more accessible model of AAC intervention that could help more families receive early intervention services and/or provide communication strategies while they are waiting to receive formal services.

For parents with autistic children with communication support needs, what is the effect of the POWR online module on the following:

(1a) Parent communication opportunities and parent responsiveness?
(1b) Child communication when paired with an SGD?
(1c) Parents’ implementation fidelity of the POWR strategy?

Question (1a) is the primary research question and questions (1b) and (1c) are more exploratory.

There is a need for social validity data for AAC interventions in the field [28], This study went beyond this need and deeply explored the impact of the POWR + SGD intervention on the everyday lives of children and their families. Additionally, we examined the feasibility of the intervention regarding reducing barriers and the acceptability of implementation. Therefore, this study also asks the following questions:

(2a) Does this intervention impact family and child functioning?
(2b) Do families find the intervention to be acceptable and feasible?

To address question (2a), the FIATS-AAC38 (The Family Impact of Assistive Technology Scale for Augmentative and Alternative Communication) [29] short-form survey was administered to parents pre- and post-intervention. Question (2b) was answered via interviews with the parents during and after the study. All study procedures described were approved by the Institutional Review Board at the first author’s university prior to recruitment.

## 2. Method

### 2.1. Participants

Participants were recruited through contacts provided by two universities in Southern California. Additionally, flyers were posted online in autism groups on social media. Parents were sent basic information about the study and asked to contact the researchers if interested. Researchers contacted interested parents via a phone call to determine eligibility and to answer questions about the study, requirements to participate, rights, and data collection. If families were eligible and wished to participate, they were sent consent forms, demographic questions, the Inventory of Potential Communicative Acts (IPCA), and the FIATS-AAC38 to complete [29]. Prior to the start of the study, parents completed The Inventory of Potential Communicative Acts (IPCA) [30]. Eight families were contacted and three were found eligible for participation. Two families consented and participated in the study.

The inclusion criteria for the parents were as follows: (a) no previous AAC parent-implementation training, (b) no training in parent responsiveness, (c) basic computer skills, and (d) being the parent or primary caregiver to a child that meets the child inclusion criteria. The child inclusion criteria were that the child (a) has or is suspected to have autism; (b) is either nonspeaking or has limited verbal skills; (c) is between three and eight years old; (d) is not deaf, hard of hearing, or blind; (e) can use direct selection to access a tablet (using fingers).

### 2.2. Dyad A

The first parent–child dyad was the mother, Elsa, and her child Chris. Elsa is a Caucasian woman of European descent in her early forties. She and Chris live in the United States on the northern part of the east coast. She has a graduate-level education and works in the healthcare field. Chris is her only child. She does not identify as disabled or neurodivergent herself. Elsa speaks English and Serbo-Croatian to Chris at home. Elsa reports that Chris identifies as male. He is seven years and nine months old at the start of the study. Chris’ favorite things to do are playing with a penguin and owl that light up and sing, vacuuming, playing with ball/catch, reading picture books with his mom, dancing to his favorite music, and enjoying snack time, particularly pouches. Chris is warm and friendly. He has an intellectual disability and receives services for developmental delay and cerebral palsy. It is suspected he has autism, and he currently sees an autism specialist. He also attends speech therapy, occupational therapy, and physical therapy. Chris communicates using gestures, signs, facial expressions, and sounds. Receptive language is an area of strength for Chris. Elsa reports that she has tried real-world pictures and using sign language to promote communication at home. Before the study, Chris was on a waiting list to receive AAC services.

### 2.3. Dyad B

The second parent dyad was Sandra and her son Antonio. Sandra is in her late twenties and married. She has an associate’s degree and works as a paraprofessional in a local public middle school. She had no training using AAC or responsiveness strategies before the study. Sandra identifies as Latina and Caucasian. She reports that it might be possible that she is neurodivergent or has a disability; however, she does not have a diagnosis. She, her husband, and two children reside in the southeastern region of the United States. She and her husband speak both Spanish and English with their children. Antonio is three years and zero months old at the start of the study. He loves counting, letters, and singing his favorite parts of songs. Sandra shared that her friends and colleagues comment on his intelligence. Antonio is autistic and receives early intervention services for speech, occupational therapy, and physical therapy. He does not have an intellectual disability. Antonio communicates through gestures, screams, and some words. He often speaks using gestalt forms or echolalia. He has difficulty answering questions and telling people what he needs. According to Sandra, the SLP on Antonio’s early intervention team suggested that AAC could be a good fit for Antonio. Before the study, she and the team were in discussions about a device and services.

### 2.4. Research Design

The design of this study was a multiple-baseline design to measure data on parents and children during the parent-mediated AAC intervention in a naturalistic setting. Dependent variables are parent communication opportunities, responsiveness, and children’s communicative acts. Parent communication opportunities were the central dependent variable that was used for determining when to move participants through conditions.

### 2.5. Setting and Materials

The POWR training modules, including preparing the AAC, were completed by participants in their homes. Baseline and intervention sessions took place in the dyad’s home over Zoom. The materials used were toys or activity items the child normally uses at home, a computer or tablet for Zoom, and another tablet as an SGD. The SGD provided was an Amazon Kindle table with Coughdrop, an open-source AAC application [27]. The recordings were stored on a password-protected cloud application to which only the lead author had access. All data were redacted of names and other identifying information and stored under participant IDs. All participants consented for their data to be retained and used for conferences and teaching.

### 2.6. Procedures

#### 2.6.1. Baseline Phase

During baseline, data were collected on the three dependent variables—parent communication opportunities, parent responsiveness, and child communicative acts. The baseline sessions were all 10 min in length. Dyads were given the instruction, “Play as you normally play”. Each dyad would move out of baseline when the previous participant’s data became stable.

#### 2.6.2. Parent Training

After baseline, parents were emailed log-on information and a link to the online modules. In addition, they were given a tablet and directions to set up Coughdrop [27] an open-source SGD app. The directions outlined how to set up the app and create a core word board on the tablet. Both parents completed the modules and set up the SGD within two weeks. After parents completed the modules, they were locked before the intervention phase of the study to address concerns about internal validity.

#### 2.6.3. Intervention Phase

After the parents completed the modules, the intervention phase took place via telepractice. During the intervention phase, parents implemented what they learned during the training with their children in a naturalistic setting in their homes. Parents were told, “Use the POWR strategy with your child”. This instruction meant they needed to follow all the steps of POWR—(1) prepare the activity and AAC; (2) offer an opportunity for communication; (3) wait for the child’s communication; and (4) respond to the child’s communication. Furthermore, in order to follow step one to fidelity, they had to select the activity and ensure the SGD core board included appropriate vocabulary. After providing the prompt, the study staff turned off their video and sound so that they could not be seen or heard by the dyad. Then, they observed the interaction for 10 min. During the 10 min of implementing the POWR strategy, the dependent variables were counted. After 10 min elapsed, the study staff turned the camera and sound on and told the dyad the session was complete. Directly after the session, the videos were transcribed and coded line-by-line using CLAN (Computerized Language ANalysis) [31] by the first author. Douglas and colleaguesstudy manual’s instructions and definitions of codes were utilized [3]. Videos were chosen at random and coded for interobserver agreement by the last author who was blind to the video condition.

#### 2.6.4. Maintenance Phase

Maintenance sessions took place two and four weeks after the intervention phase was completed. During the maintenance phase, parents were told, “Use the POWR strategy with your child”. Dyad A completed the study but was unable to participate in the maintenance phase due to unforeseen circumstances. Therefore, we do not know if there was any sustained impact of the intervention for Dyad A.

## 3. Results

### 3.1. Response Measurement and Interobserver Agreement

For the first research question, communication opportunities provided by the parent and parent fidelity were the dependent variables. Parent communication opportunities were the central variable used to make decisions about the number of sessions for the study. For the second question, child communication was the dependent variable. The final exploratory research question, parent fidelity, used multiple variables to start to evaluate parent fidelity based on each component of the POWR strategy. Douglas and colleagues’ variable definitions and coding were utilized [4]. The main dependent variable in the study was the communication opportunities provided by parents. These were defined as comments, choices, or questions directed at the child, with a wait time of at least 5 s or a response from the child within 5 s. Additionally, two secondary dependent variables were measured. Parent responses were defined as acknowledgments (e.g., “you want the doll”) or fulfilling the child’s communicative intent (e.g., the child asks for a doll and the parent provides it) within 5 s of the child’s communication, using either verbal or nonverbal means. Child communication was defined as any message sent from the child to the parent using speech, gestures, or AAC (e.g., the child looks at the parent while communicating and uses sign language), including both responses and initiations. This communication ended when the parent responded or after 3 s had passed.

Communication opportunities provided by parents, parent responsiveness, and child communication acts were counted during the intervention and directly after from the video recordings. Videos were chosen at random and coded by one independent, blinded observer—the last author. There is one video per session for each participant: eight videos for Dyad A and fourteen for Dyad B. Videos were selected from each phase, i.e., 40% baseline and 40% intervention. Interrater reliability was 85% for parent communication opportunities (range = 75–93%) and 90% for parent responsiveness (range = 83–95%). Data for collection ended early for Dyad 1 due to needing surgery, so we were unable to collect maintenance data for this dyad.

### 3.2. Parent’s Communication Opportunities

**Figure 1:**
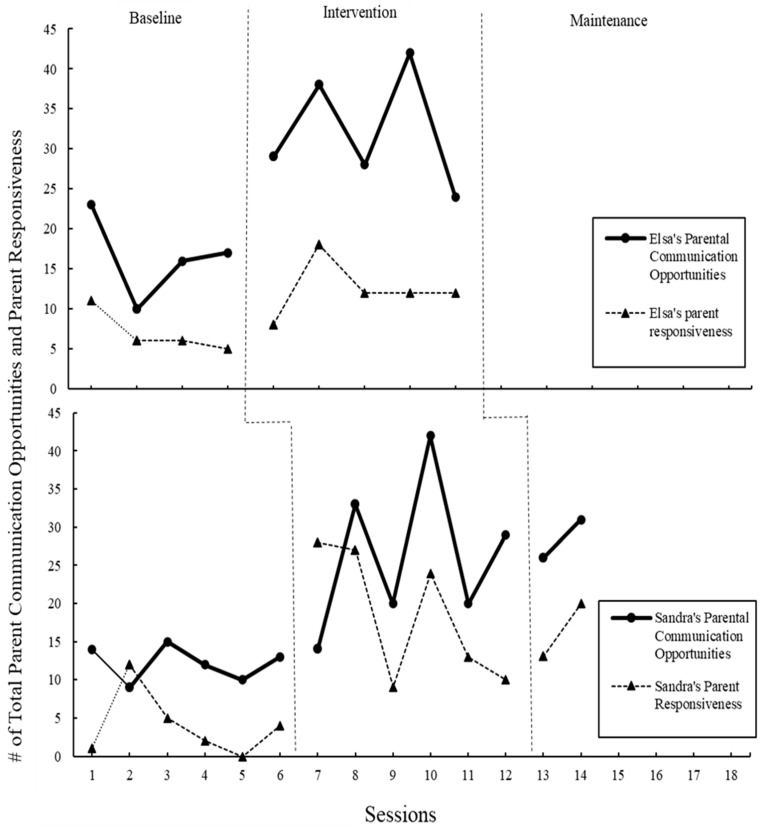
Total number of parent communication opportunities provided by Elsa and Sandra and total of parent responses. All sessions were 10 min.

As seen in Figure 1, during baseline, Elsa had a mean of 16.5 for parent communication opportunities (range = 10–23). During the intervention phase, the mean increased to 32.2 (range = 24–42) for parent communication opportunities (see Figure 1). During baseline, Sandra had a mean of 12.2 for parent communication opportunities (range = 9–15). During the intervention phase, the mean increased to 26.33 (range = 14–33) for parent communication opportunities. During maintenance, at two weeks and four weeks post-intervention, the mean increased slightly to 28.5 (range = 26–31). The range and visual inspection of the data reveal that parents became stronger at providing communication opportunities as they had more exposure to the intervention and practice using the strategy.

### 3.3. Parent Responsiveness

Elsa had a mean of 7 for parent responsiveness (range = 5–11) during baseline, as seen in Figure 1 above. During the intervention phase, the mean increased to 12.4 (range = 8–18) for parent responsiveness. Sandra had a mean of 4 for parent responsiveness (range = 0–12) during baseline. During the intervention phase, the mean increased to 18.5 (range = 9–28) for parent responsiveness. During maintenance, the mean decreased slightly to 16.5 (range = 13–20).

### 3.4. Child Communicative Acts

**Figure 2:**
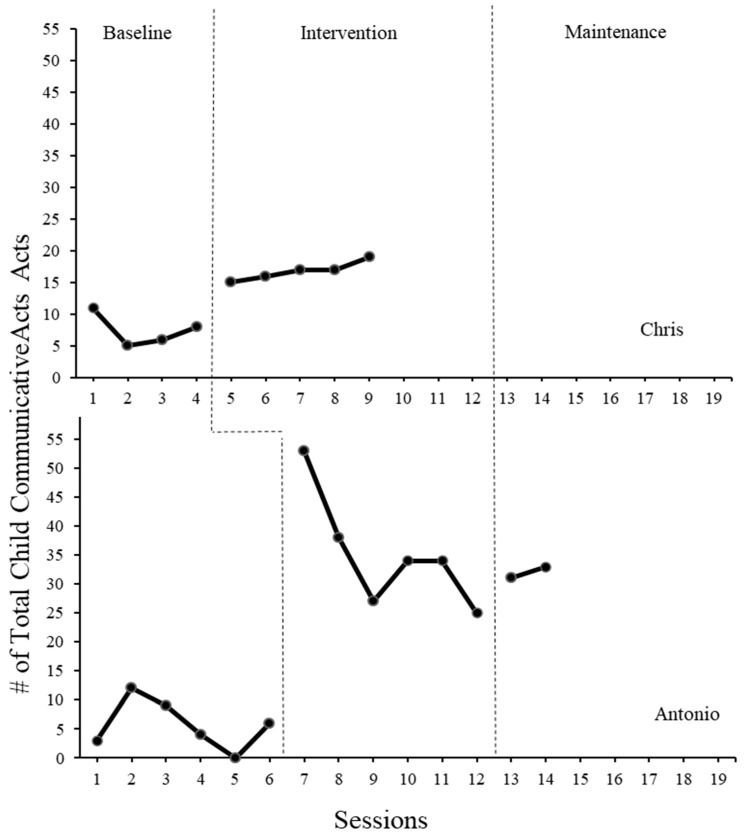
Total number of communicative acts completed by Chris and Antonio during baseline and parent implemented AAC intervention sessions. All sessions were 10 min.

Figure 2 shows that during baseline, Chris had a mean of 7.5 communicative acts (range = 5–11). During the intervention phase, the mean increased to 16.8 (range = 15–19) communication acts. During baseline, Antonio had a mean of 5.7 communicative acts (range = 0–12). During the intervention phase, the mean increased to 35.2 (range = 25–53) communicative acts. During maintenance, the mean dropped slightly to 32 (range = 31–33). The types of communication varied for each participant (see 12).

### 3.5. Parent Fidelity

**Figure 3:**
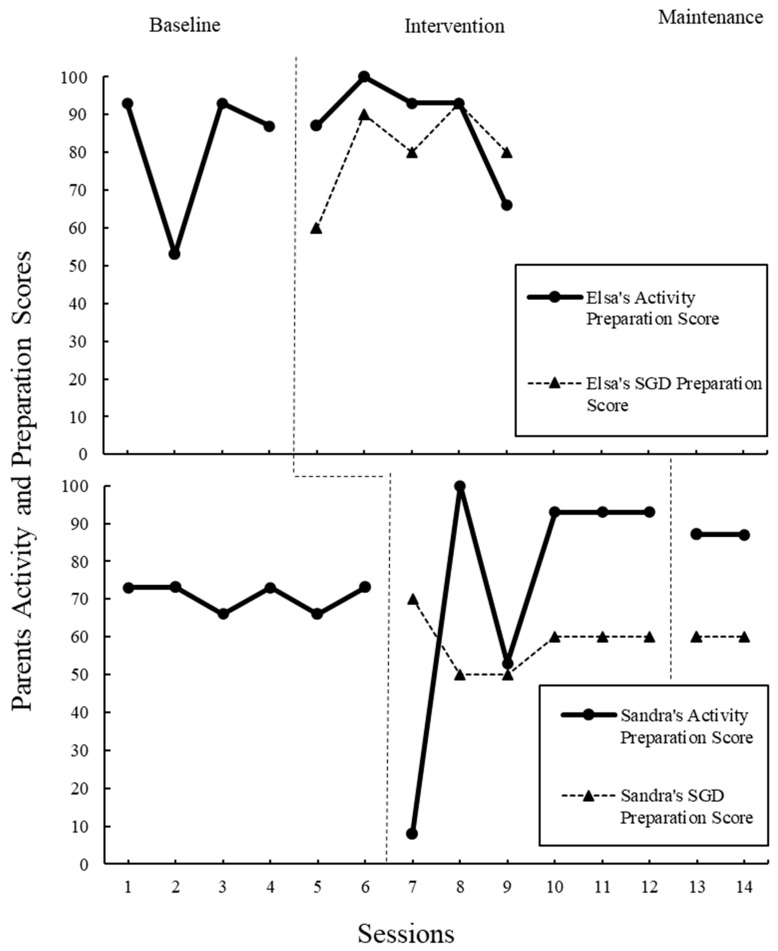
Parents’ preparation scores out of 100 possible areas of activity and SGD preparation. High scored indicate fidelity to POWR’s “P”, prepare the activity and AAC.

In addition to the variables outlined above, videos were coded for other core elements of the POWR strategy to examine how and if parents were reaching fidelity through the modules alone. To examine step 1, “P” for “Prepare the Activity and AAC”, videos were rated using a rating scale adapted from Douglas) and colleagues (Figure 3) [3]. The original scale was used for parents to self-rate how well they could use the POWR strategy in the clinic prior to the study. The one similarity between the scales is the type of questions. The scale for the current study was created to measure parent fidelity during study implementation. In addition, missed opportunities due to waiting time, count of directions given, and count of the time parents modeled the AAC were also gathered. These acts, in addition to parent communication and parent responsiveness, provide insight into how parents are implementing steps two to four, “OWR”. All instances were coded and counted during transcription using the definitions from Douglas and colleagues original 2017 study [3]. The fidelity scale was used to help analyze the data and understand the impact of fidelity on the outcome variables.

To answer the question of parent fidelity for using POWR, each element of the strategy was considered. For “P”, prepare the activity and the AAC device, parents were rated on preparing the activity and preparing the AAC device (see Table A1). These rating scales were adapted from Douglas’s original parent self-rating scale [3]. Intervention and maintenance scores were compared with the baseline (see Figure 3). Elsa received high scores for preparing the activity across all phases of the intervention. Additionally, Elsa was quick at learning to prepare the SGD during the intervention phase with an average score of 88% after taking the modules. Sandra showed improvement in preparing activities after taking the modules. Although she did not pick up the skill as quickly, her SGD preparation became stable at 60% at the end of the intervention phase.

**Figure 4:**
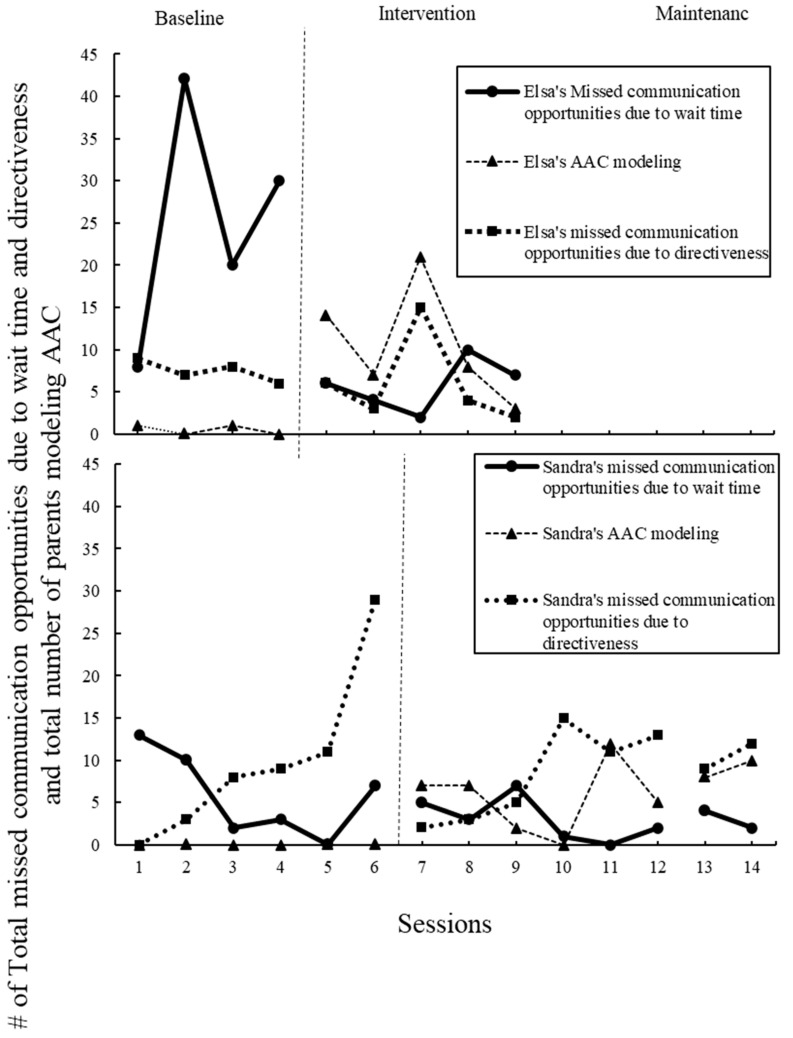
Parents’ total number of missed communication opportunities due to wait time, total number of missed communication opportunities, directiveness, and total number of parents modeling AAC. Missed wait time shows lack of fidelity to “W”, wait, in POWR. AAC modeling, along with communication opportunities, indicated fidelity to “O”, offer, in the POWR strategy.

The second step of POWR, “O”, which offers an opportunity for communication, was explored by looking at the increase in parent communication opportunities, the central variable (see above and Figure 1), and the decrease in missed communications due to directiveness (see Figure 4). In the modules, parents were instructed on how to give communication opportunities, which included the instruction that giving directions does not elicit communication. Both parents increased parent communication opportunities; however, no change in the number of directions given was observed across all phases of the study.

Step three, “W”, waiting for the child’s communication was investigated by counting the number of missed communication opportunities due to wait time. In the modules, parents were instructed to wait at least five full seconds after providing a communication opportunity to the child. Elsa’s missed communication opportunities decreased from an average of 20 at baseline to 6 during the intervention phase. Sandra’s missed opportunities due to waiting time were relatively low at baseline, with an average of 6, an average of 3 during intervention, and an average of 3 during maintenance.

Fidelity to the final step, “R”, response to child communication was measured by counting parent responses and comparing pre- and post-baseline. Both parents increased in their total responsiveness counts throughout the study (see Figure 1).

Last, although not a letter in the POWR acronym, modeling using the AAC is explicitly taught in the modules. Therefore, any time the parent modeled using AAC, either the SGD, sign, or another type, was counted (see Figure 4). At baseline, Elsa modeled sign averaging 0.5 per session, and during intervention, Elsa modeled both the SGD and sign averaging 11. At baseline, Sandra did not model AAC. During the intervention, her modeling averaged 6, and during maintenance, 9.

### 3.6. Social Validity

To determine the functional impact of the parent training intervention, parents completed the FIATS-AAC38 [29], the abbreviated version of the FIATS-AAC (Family Impact of Assistive Technology Scale for Augmentative and Alternative Communication). Parents were also interviewed after sessions and via written input three months after completing the study to obtain insights into the acceptability and feasibility of the modules in combination with an SGD.

#### 3.6.1. Family Impact of Assistive Technology Scale for Augmentative and Alternative Communication

The Family Impact of Assistive Technology Scale for Augmentative and Alternative Communication (FIATS-AAC38) is a shortened version of the FIATS-AAC, designed to measure the impact of AAC interventions on children and their families. The FIATS-AAC38 is a reliable parent-reported questionnaire that identifies child and family functioning in seven domains—behavior, education, face-to-face communication, self-reliance, social versatility, security, and supervision. These are domains that are likely to be affected by AAC interventions for individuals ages 3 to 18 years. These domains are crucial because they provide a comprehensive view of how AAC interventions affect various aspects of a child’s life and their family’s well-being.

For Dyad 1, Chris’s social versatility improved, while face-to-face communication and supervision improved slightly. However, behavior and education received lower scores post-study. This is indicative that the use of an SGD had very little impact in everyday life for Chris outside of the direct aims of improved social communication. For Dyad 2, however, the findings indicate unanimous improvement for Antonio—from the highest being a full 1.6 points higher (education), with the only decrease being social versatility (−0.8). This suggests that Antonio’s behavior in the classroom, in different social situations, and with his parent/s seems to have improved post-intervention.

#### 3.6.2. Interviews

Parents were interviewed after the completion of the study to further understand the acceptability and feasibility of the intervention. Elsa shared, “The POWR modules were easy to access at my convenience and use at home with Chris. I have found that the key of a successful therapeutic modality is the ability to execute the task at home. The modules were very accessible, and [the] format was user friendly”. Both parents report that they will be implementing the strategies learned in their everyday lives with their children. Sandra independently stated in a session that she plans to teach the strategy to other family members. In addition, Elsa shared that she appreciated the systematic approach of the POWR and she felt the intervention in combination with the SGD helped her understand her role as a communication partner with her child. Three months after the study was complete, Elsa reported that she was still using the strategy and device with her child. Her child, Chris, is now receiving AAC services from a speech-language pathologist at school and is using a Switch AAC. Overall, the parents found the modules and implementation feasible and acceptable, and neither parent mentioned any difficulties or hardships in implementing the intervention with their child.

## 4. Discussion

### 4.1. Central Research Question: Parent Communication and Responsiveness

The overall goal of the POWR modules is to teach parents how to provide communication opportunities to their children. In previous studies examining the POWR strategy’s effectiveness, this was one of the central variables [3,4]. After the introduction of the POWR strategy and the SGD, the study found an increase in parent communication opportunities. The findings are similar to the previous studies that used the POWR strategy alone. Overall, the POWR modules appear to positively impact the communication opportunities provided by the parent during play and activities. Although there was a large increase, the data do not appear to be stable; however, it never decreased to baseline levels. The lack of stability could be due to the parents using a different set of toys or activities during most sessions. The modules encourage parents to find activities and play that elicit the most possible communication. Both parents independently chose to experiment to find vocabulary and activities that elicited the most communication from their children during the study. Although it was important to the process of learning to use the strategy, it does pose a risk to internal validity. Although the data indicate a significant increase, stability issues have been noted, likely due to the variability in activity types. Consistently choosing the same or similar activities in each session could have potentially mitigated this variability.

### 4.2. Research Question Two: Child Communication Acts

The parent’s use of the POWR strategy after only taking the online modules appears to have an impact on child communication. Although parents were not using the strategies perfectly for fidelity and in many different play contexts, both child participants’ communication increased. It appears that the type of activity selected has a large impact on child’s communication. For example, across dyads, reading a book was an activity that did not elicit much communication (see Table 1).

Chris’s communication opportunities increased steadily and remained stable in the intervention phase. However, Antonio had a drastic increase in communication followed by a decrease. At two weeks and four weeks post-intervention, Antonio’s communication remained higher than pre-intervention. Both children stayed well above their baseline levels once their parents began using the POWR strategy with them. The type of communication acts differed for each participant. Chris’s communication was limited to gestures, pointing, and signs at baseline. After the introduction of the SGD and the POWR strategy, he began to use the SGD device as modeled by his mother. He requested things, invited his mother to have a turn, and made choices using the SGD. This was his first introduction to using an SGD. Antonio had a very different communication profile at baseline. He communicated with single words and by showing objects he wanted or did not want to engage with. During the POWR strategy and the introduction of the SGD, he used the SGD 20 times across 6 sessions. Additionally, his spoken communication increased. During the baseline, his spoken utterances averaged 5 per session. During the intervention, he averaged 31 per session. At maintenance, he averaged 29 per session. This finding is consistent with previous research that the introduction of AAC can lead to increased spoken communication for young children with developmental disabilities 3 [32,33]. The implementation of the SGD and POWR strategy resulted in enhanced child communication, aligning with prior studies and reinforcing the case for the intervention’s effectiveness.

### 4.3. Research Question Three: Parent Fidelity

Overall, parents became more proficient at the POWR strategy as the study progressed. Even though both mothers’ implementation of each step of POWR improved and they were communicating better with their children, it appears that more training, such as parent coaching or in-clinic training after taking the modules, would help parents reach fidelity [34,35]. However, given the improvements and the social validity findings, the POWR strategy modules appear to be a good first step for families.

### 4.4. Implications

#### 4.4.1. Needs of Parent and Child: Are the Online Modules Alone Enough?

The POWR modules and parent intervention alone would not be enough communication and AAC support for many autistic children and their families. AAC users and their families need access to speech-language pathology, AAC training, AAC assessment, and AAC supports across the lifespan [36,37]. However, the diagnostic journey is long and full of waiting for services for autistic children and their families to even get access to any services [38], Online modules and an SGD are a promising solution for a future intervention that uses very few resources while waiting for speech-language pathology services and an AAC assessment. Evidence suggests that adding coaching would increase efficacy; however, having an option that does make an impact while using very few resources is a novel and needed approach to provide support to children and their families as soon as possible.

The results from this study are promising and the POWR strategy is ideal for families awaiting services or those who have services but there is a home–clinic–school communication divide. There is a need for more research that investigates early support during the waiting time of the diagnostic journey—the vital developmental years when early intervention has the greatest impact. There is an opportunity to investigate the use of the POWR strategy with families who are already receiving AAC services and need to bridge the gap between home, school, community life, and clinic.

The POWR strategy is a framework for using AAC with a child during an activity. For this study, the only instruction for how to use the AAC was written instructions that were not a part of the module. In future research, some basic SGD instructions need to be a part of online modules. There is a potential to create new modules that give SGD instruction and a play strategy and framework for using an SGD for autistic children specifically. The POWR strategy is designed for all children with complex communication needs and is not specifically targeted for autism.

#### 4.4.2. Child Communication Acts and Fit for Autistic Populations

The findings of this study and the previous study of parents using the POWR strategy with their autistic children [5] together are the beginnings of a body of evidence that supports the expanded use of this intervention, originally created broadly for children with communication support needs. However, there is the potential for improvement to the strategy to fit autistic populations who are non-speaking or have communication support needs.

There is a strong body of evidence that parent responsiveness, particularly verbal responsiveness, improves social communication and language outcomes for autistic children [23,25,39]. In the POWR modules, parents are taught to respond to child communication. However, there is an opportunity to add to the modules and give a few examples of richer parent responsiveness using specific praise or an expansion.

Another change to consider for autistic or potentially autistic children would be to explicitly teach following the child’s interest during play and offering expansions—even if the play can seem silly or not done the right way. For example, Antonio was completing a sticker book activity with his mom, Sandra. He wanted to put the sticker upside down and in “wrong” places. Sandra gave Antonio the communication opportunity, “Where does that sticker really go?” and began to give directions when Antonio did not play “correctly”. Similar scenarios happened with both dyads throughout the study, which is in line with previous research on mothers of autistic children who tend to be more directive [40].

Last, the modules should teach parents activities that would be the most ideal for autistic children. In sessions where parents used toys and symbolic play as the activity, children were the most communicative. Additionally, when parents chose activities that most interested their child, communication, and communication opportunities were similarly high. For Antonio, this was any game or activity about numbers, and for Chris, this was playing with a light-up and dancing penguin. Reading books gave the lowest scores for all dependent variables across both dyads. Therefore, the modules could recommend parent activities that utilize toys and symbolic play and encourage parents to follow their children’s interests.

#### 4.4.3. Limitations and Future Directions

The main limitation of this study was the small number of participants (two dyads). Originally, three dyads were recruited for this preliminary study, but one of the dyads (Dyad C) decided not to participate prior to starting data collection. This happened during baseline data collection for Dyad A, and since data needed to be collected concurrently, there was not sufficient time to recruit a new participant. The research team was unsuccessful in recruiting another dyad in time during data collection. The small number of participants served the purpose of this study in determining its feasibility. This study also had problems with attrition, and the researchers were unable to collect maintenance data for Dyad A due to a medical emergency. Although we have data on the initial impact of the intervention over several weeks, we do not have any knowledge of the long-term impact of the intervention for Dyad A. As the next step, this study will be expanded. The aim is to recruit a diverse sample of at least five dyads to ensure robust external validity while accounting for attrition.

Additionally, Chris and Antonio, while both meeting the inclusion criteria, have different profiles. Chris has suspected autism but is diagnosed with an intellectual disability and cerebral palsy while Antonio’s only diagnosis is autism. Furthermore, they came from different racial and economic backgrounds.

The results from this indicate that the remote POWR AAC training is a good addendum to in-person training that has accessibility barriers. The results from this study suggest that early interventionists should consider telehealth parent training as part of their typical practice. However, since the module was pre-recorded, there was no interactive coaching and feedback given to the parents and children, which could further help the parents become more accustomed to using AAC with their child. Collaborative coaching with feedback given by an AAC professional is key to AAC partner training, an evidence-based practice [41], With long waitlists and SLP shortages nationwide, there is a clear need to understand ways of providing young children with communication support needs due to developmental disabilities with accessible and feasible parent-mediated AAC. Future studies should explore using the modules with coaching. Additionally, all studies examining the POWR strategy have coded child communication acts broadly (see Table 2). It would be interesting to further analyze the complexity and richness of child communication in further detail.

## 5. Conclusions

There is a great need to alleviate barriers and provide AAC to autistic children during early childhood when AAC interventions have the greatest impact on both verbal and aided communication skills [10]. Asynchronous parent training utilizing online communication modules in combination with an SGD is a promising solution. The strategy appears to improve parent responsiveness and communication opportunities, which may lead to long-term improvements in child communication and social engagement. Additionally, the parents found the intervention to be accessible and feasible. Based on the results of the FIAT-AAC38, the parents introducing the child to the SGD made positive impacts on the child and overall family functioning for the two study participants. There is a need to replicate this preliminary study with more participants as a larger single-case design or a randomized controlled trial. Additional research should explore whether the addition of in-person and/or telehealth coaching improves both parent and child outcomes, and the strategies should be used in more diverse samples of children with ASD with a broad range of communication profiles. This study is a step forward in finding innovative ways of providing training and access to families of young children with ASD for communication and AAC who are waiting for services or have geographic or financial barriers to accessing services. Finally, our findings should influence disability service policy so that young children and their families may more easily access SGDs through a loaning library and be provided free access to online training prior to formal AAC evaluation and the process to obtain funding.

## Figures and Tables

**Table 1:** Activities chosen per session.

Session	Elsa and Chris	Sandra and Antonio
Baseline		
1	Toys, light-up penguin, ball	Bus, letter popsicles, Lego blocks
2	reading books	Eggs with shapes inside, Legos
3	Toys, ball, light-up penguin, owl	Sticker books
4	Toys, light-up penguin, owl	Legos
5	—	Blank dominos
6	—	Zipper and buckles/sticker book
Intervention		
1	Cars + light-up penguin	Truck + letter popsicles
2	Steering wheel toy, ball, and snacks	Number cards
3	Dump truck +penguin	Reading picture books and toy cars
4	Penguin + cars	Marker + paper, writing letters
5	Penguin, cars, reading	Marker + paper, writing numbers
6	—	Marker + paper, writing
Maintenance		
1	—	Blocks + cars
2	—	Jenga blocks

**Table 2:** Types of communication acts for Chris and Antonio.

	Verbal	AAC-SGD	Sign Language	Point/Gesture	Object
B1 Chris	0	0	2	5	4
B2 Chris	1	0	0	1	3
B3 Chris	0	0	1	0	5
B4 Chris	0	0	3	0	5
I1 Chris	0	8	1	2	4
I2 Chris	0	7	1	2	6
I3 Chris	0	12	0	2	3
I4 Chris	0	7	1	2	7
I5 Chris	0	10	2	4	3
B1 Antonio	3	0	0	0	0
B2 Antonio	11	0	0	0	1
B3 Antonio	7	0	0	0	2
B4 Antonio	4	0	0	0	0
B5 Antonio	0	0	0	0	0
B6 Antonio	5	0	0	0	1
I1 Antonio	50	3	0	0	0
I2 Antonio	35	2	0	0	1
I3 Antonio	11	11	0	4	1
I4 Antonio	30	3	0	1	0
I5 Antonio	34	0	0	0	0
I6 Antonio	24	1	0	0	0

Note. B1–6 are baseline sessions and I1–6 are intervention sessions.

## Data Availability

The data presented in this study are available on request from the corresponding author due to privacy.

## Appendix A

**Table A1:** Rating scale to score parents’ preparation.

Name of Participant:					
Session number:					
Prepare the activity and AAC Rating	5	4	3	2	1
Is the activity motivating for the child?	Strongly Agree	Disagree	Neutral	Disagree	Strongly Disagree
Is the activity developmentally appropriate?	Strongly Agree	Disagree	Neutral	Disagree	Strongly Disagree
Does the activity allow opportunities for communication?	Strongly Agree	Disagree	Neutral	Disagree	Strongly Disagree
ACTIVITY SCORE					
Is the selected vocabulary aligned with the activity?	Strongly Agree	Disagree	Neutral	Disagree	Strongly Disagree
Functional?	Strongly Agree	Disagree	Neutral	Disagree	Strongly Disagree
SGD PREP SCORE					
